# Five Decades of Pancreas Transplantation at the University Hospital of Zurich—A Story of Continuous Improvement and Success

**DOI:** 10.1111/ctr.70368

**Published:** 2025-11-05

**Authors:** Michael C. Frey, Sandro Hügli, Olivier de Rougemont, Kerstin Hübel, Thomas Schachtner, Elena Rho, Lukas Weidmann, Roger Lehmann, Jakob Nilsson, Lukas Frischknecht, José Oberholzer, Fabian Rössler

**Affiliations:** ^1^ Department of Surgery and Transplantation University Hospital Zurich Zurich Switzerland; ^2^ Department of Nephrology University Hospital Zurich Zurich Switzerland; ^3^ Department of Endocrinology University Hospital Zurich Zurich Switzerland; ^4^ Department of Immunology University Hospital Zurich Zurich Switzerland

**Keywords:** chronic kidney disease, end‐stage renal failure, insulin‐free survival, pancreas transplantation, simultaneous pancreas and kidney transplantation, type 1 diabetes mellitus

## Abstract

**Introduction:**

Pancreas transplantation (PT) represents the gold standard in patients with type I diabetes mellitus (T1D) and end‐stage kidney disease (ESKD). We aimed to describe the evolution of PT at the University Hospital of Zurich from 1973 to 2023, and to analyze differences in patient and graft survival within different eras of surgical technique.

**Methods:**

This study represents a retrospective analysis of all primary PT performed at the University Hospital Zurich between 1973 and 2023. According to differences in surgical technique, we defined five different eras of PT.

**Results:**

Overall, 280 primary PT were performed. Overall survival at 1, 5, and 10 years improved from 73.5%, 51%, 44.9% in Era 3, to 69.7%, 60.6%, 51.5% in Era 4, and 95.8%, 95.4%, 82.1% in Era 5, respectively (*p* < 0.001). Insulin‐free survival after 1, 5, and 10 years was 34.5%, 25.5%, 16.4% in Era 3, 41.5%, 34.1%, 31.7% in Era 4, and reached 85.5%, 78%, 64.5% in Era 5 (*p* < 0.001), respectively.

**Conclusion:**

Enhanced surgical techniques and improvements in immunosuppression helped to overcome many obstacles that hampered the early days of PT, evolving PT into a safe and highly efficient procedure. PT represents the treatment of choice for patients with T1D and ESKD.

**Abbreviations**
ATG: anti‐thymocyte globulin
CCI: Comprehensive Complication Index
CIT: cold ischemia time
CKD: chronic kidney disease
DBD: donation after brain death
DCD: donation after cardiovascular death
ESKD: end‐stage kidney disease
GFR: glomerular filtration rate
HbA1c: hemoglobin A1c
IGL: Institution Georges Lopez
IQR: interquartile range
PAK: pancreas after kidney
PT: pancreas transplantation
SD: standard deviation
SPK: simultaneous pancreas and kidney transplantation
T1D: type 1 diabetes mellitus
UNOS: United Network for Organ Sharing

## 1 Introduction

Pancreas transplantation (PT) can achieve long‐term insulin independence, prolonging overall survival and improving quality of life [1, 2, 3]. For selected patients with type 1 diabetes mellitus (T1D), it represents the only definitive long‐term treatment that restores glycemic control, minimizes the risk of severe glucose dysregulation, and prevents or halts the progression of secondary diabetic complications, most importantly, chronic kidney disease [4, 5].

The world's first successful PT was performed in 1966 by William Kelly and Richard Lillehei at the University of Minnesota, where a duct‐ligated segmental pancreas graft was transplanted, simultaneously with a kidney from a deceased donor [6]. The first PT in Europe was followed in 1973 by Felix Largiadér in Zurich, Switzerland. Subsequently, there was a steady increase in PT worldwide in the 1970s and 1980s. The initial years of PT, however, were challenging due to high complication rates, resulting in previous success rates with limited patient and graft survival. Most graft losses resulted from technical complications like thrombosis or fistulas and resulted in high mortality rates, mostly for cardiovascular diseases [7]. Back then, PT was considered the solid organ transplantation with the highest complication rates [8, 9]. On top of the formidable technical challenges, high rejection rates, and the use of high doses of steroids further complicated the management of pancreas transplants.

Notable progress was achieved over the last decades, with decreasing technical complication rates, improved rejection rates, and subsequently improved long‐term outcomes [10, 11, 12]. Nowadays, most PTs are performed as simultaneous pancreas‐ and kidney transplantations (SPK) or pancreas after kidney (PAK) [8, 11, 12, 13]. SPK represents the gold standard for patients with T1D and end‐stage kidney disease (ESKD) [5] and provides a significant survival benefit compared with deceased‐ and even living‐donor kidney transplantation alone [14, 15, 16, 17]. Within the last two decades, long‐term patient and graft survival rates have steadily increased. Between 1990 and 2009, overall 5‐year survival improved from 75% to 90% in recipients of SPK [18]. One‐ and 5‐year pancreas graft survival rates significantly increased from 59.6% and 42.2% between 1989 and 1996 to 88.2% and 76.7% between 2004 and 2011 [11].

The University of Zurich has always played an important and pioneering role in PT. With the first PT ever performed in Europe in 1973 by Felix Largiadér, the activity now spans more than five decades, and we continue to hold a highly active program for PT with steady annual numbers and excellent outcomes [19]. In this analysis, we want to describe the evolution and progress within the different eras of PT at the University of Zurich from the very beginning until today. Over such a long period, analysis of outcomes can only be achieved by separation into different eras, focusing on differences in surgical technique.

This analysis may serve to better understand differences within the eras and the reasons for improvements over the decades. The goal is to help regain interest in PT and overcome prejudices against this precious type of transplantation. This could improve the offering of PT to more patients who could benefit from this transplantation.

## 2 Patients and Methods

We retrospectively reviewed all 280 primary PTs performed at the University Hospital of Zurich, Switzerland, over the 50‐year period, from December 1973 to December 2023. According to the vast differences in surgical techniques, we divided the 50‐year period into five different eras of PT, which are described in detail below. All PTs were SPK. Retransplantations were excluded from the analysis. No solitary PTs were included, as there were none performed at this center, except for retransplantations. The primary outcome measures were overall patient survival, insulin‐free survival, and dialysis‐free survival within the five eras. Secondary endpoints comprised differences in complications within the different eras. The local ethics committee reviewed and approved the study protocol (project number 2023‐ 01423).

### 2.1 Graft Function

A functioning pancreas graft was defined as no insulin treatment required after transplantation, and normal hemoglobin A1C (HbA1c) levels. Kidney graft function was assessed according to the Chronic Kidney Disease Epidemiology Collaboration (CKD‐EPI 2009) equation to estimate glomerular filtration rate (eGFR) [20].

### 2.2 Complications

Complications were assessed according to the Clavien–Dindo classification [21, 22]. The Comprehensive Complication Index (CCI) measures the morbidity on a scale from 0.0 (uneventful) to 100.0 (death) [23, 24], and was used to assess cumulative postoperative morbidity.

### 2.3 Immunosuppression

**FIGURE 1:**
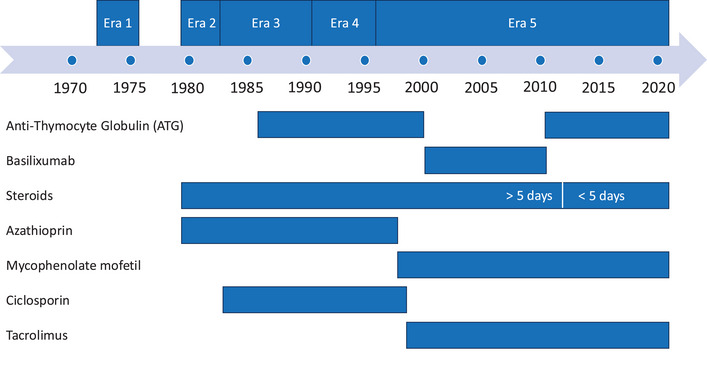
Changes in immunosuppression over time and eras.

Induction therapy was used since 1986 and was performed with anti‐thymocyte globulin (ATG) or basiliximab (Simulect). Maintenance therapy consisted of azathioprine (Imurek) and steroids; in 1983, cyclosporine was added. Mycophenolate mofetil (CellCept)/Mycophenolate sodium (Myfortic) and tacrolimus (Prograf) replaced azathioprine and cyclosporine in 1997 and 1998, respectively. Long‐term steroid treatment was administered until 2011, when the protocol was changed to an early withdrawal of steroids on Day 5. Rejection was defined as any suspicion or proof by kidney biopsy that led to specific treatment of rejection. Changes in immunosuppression are visualized in Figure 1.

### 2.4 Procurement

Almost all pancreases were procured from donors after brain death (DBD). Donors after circulatory death (DCD) were only used between 1973 and 1976 (*n* = 4), and one each in 2020, 2021, and 2023. Transport was by static cold storage in all PT, and no machine perfusion was used.

### 2.5 Statistics

Descriptive statistics was used to summarize patient and donor characteristics, surgical details, postoperative outcomes, and survival data. Categorical data was presented as absolute frequencies and percentages, while continuous variables were expressed as medians with interquartile ranges (IQR). Comparison between categorical variables was performed using the Chi‐square test. For continuous or interval‐scaled variables, a one‐way analysis of variance was used to assess differences across groups, provided the data met assumptions of normality and homogeneity of variances. Survival analyses, including overall survival, insulin‐free survival and dialysis‐free survival were conducted using the Kaplan–Meier method. Differences in survival probabilities between subgroups were assessed using the log‐rank test. To evaluate the impact of multiple factors on insulin‐free survival, a multivariable cox proportional hazards regression model was applied. Covariates included recipient age, gender, and BMI; donor age and gender; duration of preoperative dialysis; postoperative immunosuppression regimens; postoperative steroid use; cold ischemic time of the pancreas; and different surgical eras. All statistical analyses were performed using R version 4.4.2 with the packages “ggplot2” and “survival.” A *p* value of <0.05 was considered statistically significant.

## 3 Surgical Techniques and Outcome in Five Eras

**FIGURE 2:**
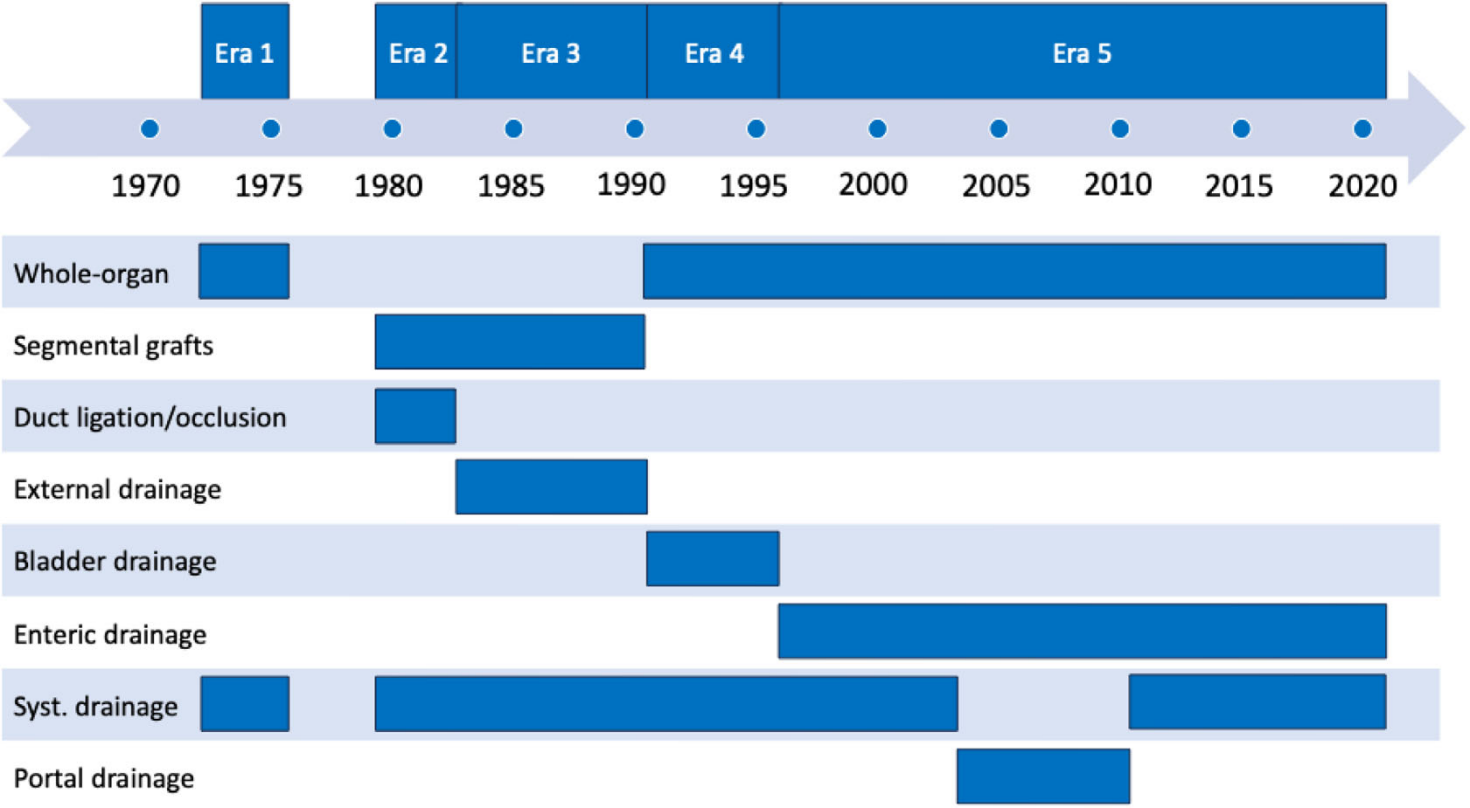
Key developments over time and eras.

**FIGURE 3:**
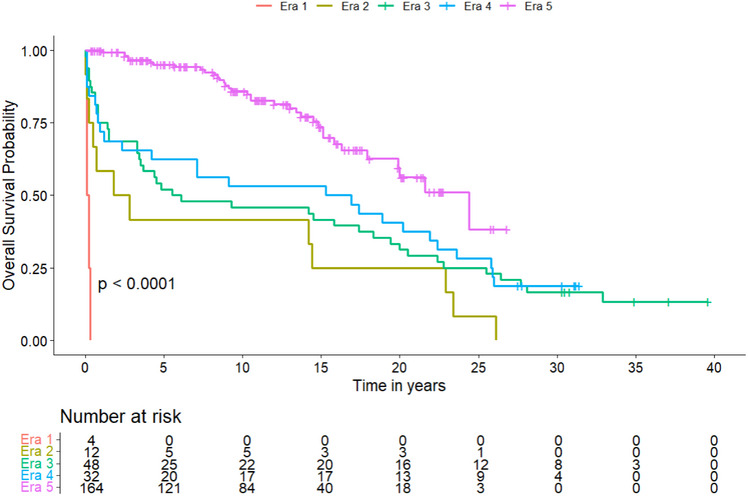
Kaplan–Meier Curve of the probability of overall survival after PT. A comparison between the different eras was performed using the log‐rank test, showing a significant difference between subgroups.

**FIGURE 4:**
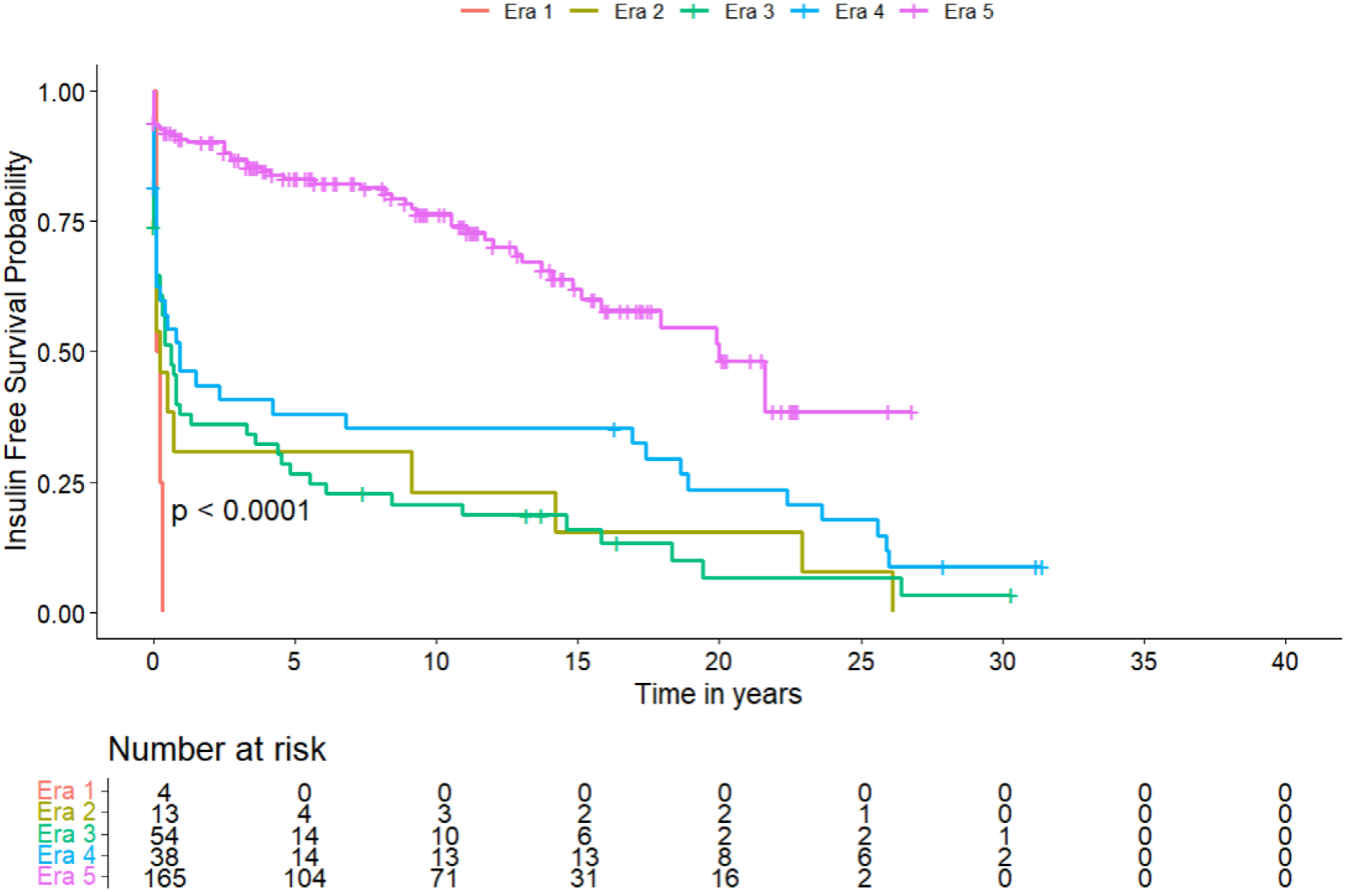
Kaplan–Meier Curve of the probability of insulin‐free survival after PT. A comparison between the different eras was performed using the log‐rank test, showing a significant difference between subgroups.

**FIGURE 5:**
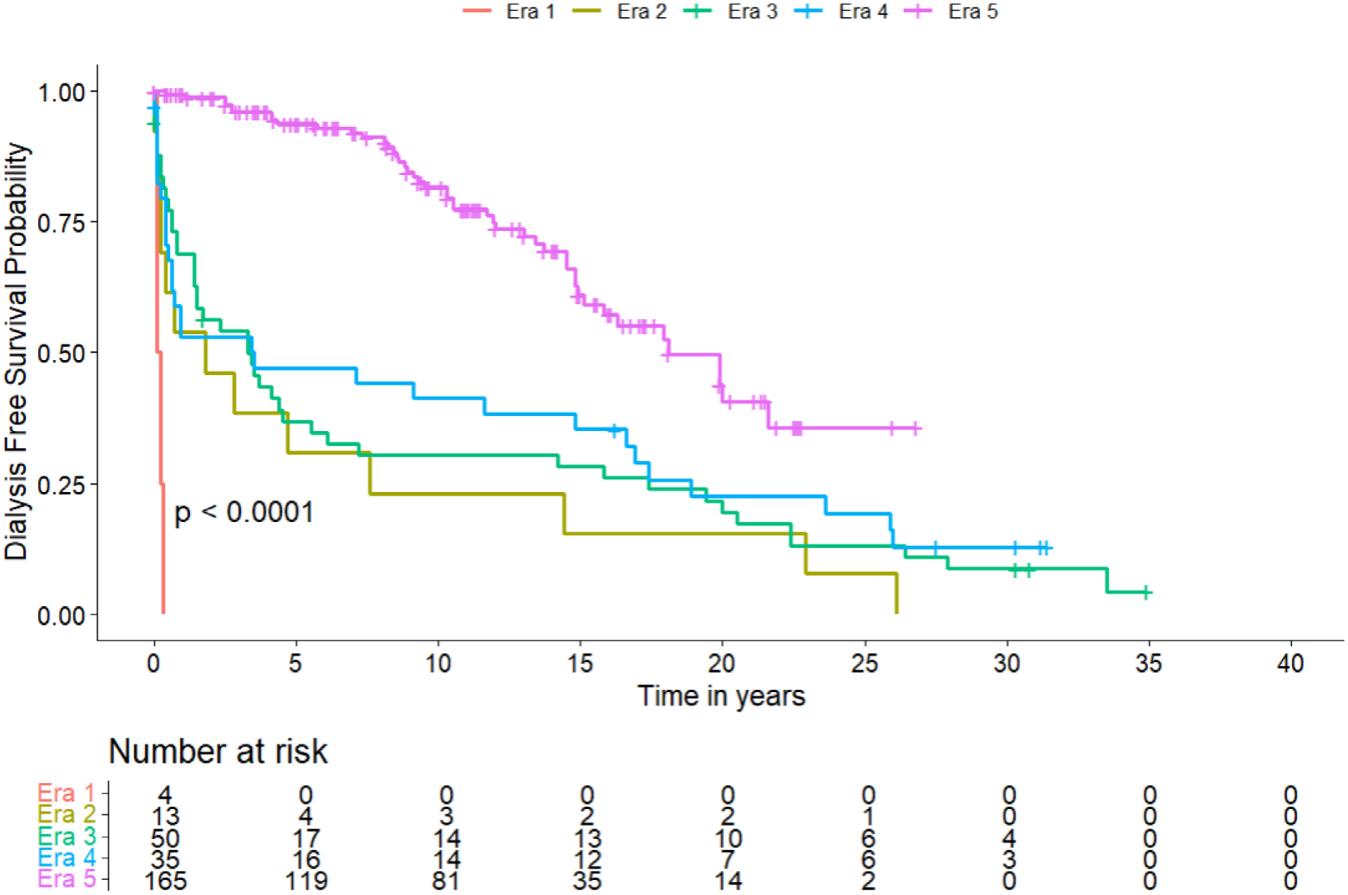
Kaplan–Meier Curve of the probability of dialysis‐free survival after PT. A comparison between the different eras was performed using the log‐rank test, showing a significant difference between subgroups.

A visualized overview of key developments is given in Figure 2. The specific numbers of transplantations per year are given in supplementary Figure 1. Details on donor and recipient characteristics within the five eras are listed in Table 1, surgical details in Table 2, and postoperative complications in Table 3. Pancreas‐specific complications are reported in Table S3. Subgroup analyses of preemptive transplantations and systemic versus portal drainage are given in Tables S2 and S4. Overall survival, insulin‐free survival, and dialysis‐free survival are shown in Table 4 and Figures 3, 4, 5, respectively. Table 5 shows the results of the multivariate analysis across Eras 3, 4, and 5.

**TABLE 1:** Donor and recipient characteristics and type of dialysis within the five eras.

Variables	Era 1 (*n* = 4)	Era 2 (*n* = 13)	Era 3 (*n* = 56)	Era 4 (*n* = 41)	Era 5 (*n* = 166)	*p* value
**Donor characteristics**						
Donor age (years)	21 [14.8–26.8]	24 [17–30.3]	26 [22–36]	35.5 [28.8–43]	32.4 [23–43]	0.001
Male donor	2 (50)	7 (53.8)	32 (58.2)	21 (58.2)	100 (69.4)	0.2
**Recipient characteristics**						
Recipient age (years)	33.5 [30.3–36]	31 [30–36]	34 [29–39.3]	37 [35–42]	42 [36–49.8]	<0.001
Male recipient	4 (100)	6 (46.2)	27 (49.1)	22 (55.0)	99 (60.0)	0.3
BMI (kg/m^2^)	NA	23.25 [22.5–25]	21.7 [20.6–23.4]	22.5 [20.9–23.5]	23.3 [21.2–25.3]	0.009
Time on the waitlist (days)	NA	NA	217 [74.8–372]	193 [54.5–483.5]	316 [136–582]	0.1
Diabetes pre‐transplant (years)	18 [18–18]	22 [18–26.5]	22 [19–26.5]	24 [20–27.3]	24 [21–30]	<0.001
**Type of dialysis pre‐TPL**						
No dialysis	0 (0)	0 (0)	8 (14.3)	8 (19.5)	47 (28.3)	<0.001
Hemodialysis	3 (75.0)	2 (15.4)	14 (25.0)	13 (31.7)	67 (40.4)	<0.001
Peritoneal dialysis	0 (0)	10 (76.9)	30 (53.6)	16 (39.0)	34 (20.5)	<0.001
Both	0 (0)	0 (0)	4 (7.1)	3 (7.3)	12 (7.2)	<0.001
Missing	1 (25.0)	1 (7.7)	0 (0)	1 (2.4)	6 (3.6)	<0.001
Duration of dialysis pre‐transplant (days)	371 [269–432]	432 [308–521.5]	465 [264.5–678.5]	525.5 [384–863.5]	601 [327–914.5]	0.1

*Note:* Continuous variables are expressed as median with IQR in []. Categorical or ordinal‐scaled variables are presented with absolute numbers (*n*) and percentages in (). Percentages may not add up to 100% due to rounding.

Abbreviations: BMI, body mass index; NA, not available; TPL, transplantation.

**TABLE 2:** Surgical details.

Variables	Era 1 (*n* = 4)	Era 2 (*n* = 13)	Era 3 (*n* = 56)	Era 4 (*n* = 41)	Era 5 (*n* = 166)	*p* value
**Surgical details**						
Operating time (minutes)	NA	NA	260 [260–260]	NA	327 [262.5–390]	0.4
CIT pancreas (minutes)	270 [225–310]	510 [315–750]	360 [180–510]	540 [433.5–711]	523.0 [442–612]	<0.001
CIT kidney (minutes)	NA	NA	NA	600 [570–780]	625 [539–755]	0.9
RT pancreas (minutes)	NA	NA	NA	NA	35.5 [31–40.8]	
RT kidney (minutes)	NA	NA	NA	NA	40 [32–47]	
**Arterial anastomosis**						0.005
CIA	4 (100)	10 (77)	43 (76.8)	35 (85.4)	157 (94.6)	
EIA	0 (0)	3 (23)	10 (17.9)	5 (12.2)	6 (3.6)	
IIA	0 (0)	0 (0)	2 (3.5)	1 (2.4)	0 (0)	
Aorta	0 (0)	0 (0)	1 (1.8)	0 (0)	2 (1.2)	
Other	0 (0)	0 (0)	0 (0)	0 (0)	1 (0.6)	
**Venous anastomosis**						<0.001
SMV	0 (0)	0 (0)	0 (0)	0 (0)	49 (29.5)	
CIV/IVC	4 (100)	13 (100)	56 (100)	41 (100)	117 (70.5)	
**Pancreas graft placement**						<0.001
Left	4 (100)	13 (100)	54 (96.4)	1 (2.4)	3 (1.8)	
Right	0 (0)	0 (0)	0 (0)	40 (97.6)	161 (97.0)	
Middle	0 (0)	0 (0)	2 (3.6)	0 (0)	2 (1.2)	

*Note:* Continuous variables are expressed as median with IQR in []. Categorical or ordinal‐scaled variables are presented with absolute numbers (*n*) and percentages in (). Percentages may not add up to 100% due to rounding.

Abbreviations: CIA, common iliac artery; CIT, cold ischemia time; CIV, common iliac vein; EIA, external iliac artery; IIA, internal iliac artery; IVC, inferior vena cava; NA, not available; RT, rewarming time; SMV, superior mesenteric vein.

**TABLE 3:** Postoperative course within the 5 eras.

Variables	Era 1 (*n* = 4)	Era 2 (*n* = 13)	Era 3 (*n* = 56)	Era 4 (*n* = 41)	Era 5 (*n* = 166)	*p* value
**Complications**						
Complications until discharge	4 (100)	11 (91.7)	55 (98.2)	39 (97.5)	117 (70.5)	<0.001
Complications within 90 days postoperative	4 (100)	12 (100)	56 (100)	39 (97.5)	141 (84.9)	0.004
CCI at discharge	100 [100–100]	44.9 [37.2–58]	57.3 [42.7–73.9]	53.1 [33.7–66.8]	20.9 [0.0–39.7]	<0.001
CCI at 90 days postoperative	100 [100–100]	44.9 [39.7–73.4]	58.4 [47.6–74.2]	53.1 [38.9–71.6]	33.6 [20.9–47.6]	<0.001
DGF kidney	0 (0)	0 (0)	4 (7.5)	3 (7.9)	10 (6.0)	0.9
Length of stay (days)	28 [24.5–69.5]	48.5 [38.5–68]	58 [45.5–78.5]	46 [29–57.5]	16 [12–24]	<0.001
Readmission within 90 days	0 (0)	6 (75)	21 (47.7)	12 (43.3)	65 (43.3)	0.4
Reoperation until discharge	2 (66.7)	9 (75)	48 (85.7)	21 (53.8)	44 (29.5)	<0.001
**Highest complication at discharge**						<0.001
CDG 0	0 (0)	2 (15.4)	1 (1.8)	2 (4.9)	49 (29.5)	
CDG 1	0 (0)	0 (0)	0 (0)	0 (0)	9 (5.4)	
CDG 2	0 (0)	2 (15.4)	3 (5.4)	7 (17.1)	40 (24.1)	
CDG 3a	0 (0)	0 (0)	3 (5.4)	7 (17.1)	18 (10.8)	
CDG 3b	0 (0)	7 (53.8)	32 (57.1)	15 (36.6)	45 (27.1)	
CDG 4a	0 (0)	0 (0)	9 (16.1)	4 (9.8)	5 (3.0)	
CDG 4b	0 (0)	1 (7.7)	2 (3.6)	0 (0)	0 (0)	
CDG 5	4 (100)	1 (7.7)	6 (10.7)	6 (14.6)	0 (0)	

*Note:* Continuous variables are expressed as median with IQR in []. Categorical or ordinal‐scaled variables are presented with absolute numbers (*n*) and percentages in (). Percentages may not add up to 100% due to rounding.

Abbreviations: CCI, Comprehensive Complication Index; CDG, Clavien‐Dindo Grading; DGF, delayed graft function.

**TABLE 4:** Overall‐, insulin‐free and dialysis‐free survival within the eras.

	Era 1 (*n* = 4)	Era 2 (*n* = 13)	Era 3 (*n* = 56)	Era 4 (*n* = 41)	Era 5 (*n* = 166)	*p* value
**Overall survival**						
1‐year	0 (0)	6 (46.2)	36 (73.5)	23 (69.7)	158 (95.8)	0.001
5‐year	0 (0)	6 (46.2)	25 (51.0)	20 (60.6)	126 (95.4)	0.001
10‐year	0 (0)	6 (46.2)	22 (44.9)	17 (51.5)	87 (82.1)	0.001
**Insulin‐free survival**						
1‐year	0 (0)	4 (30.8)	19 (34.5)	17 (41.5)	142 (85.5)	0.001
5‐year	0 (0)	4 (30.8)	14 (25.5)	14 (34.1)	103 (78.0)	0.001
10‐year	0 (0)	3 (23.1)	9 (16.4)	13 (31.7)	71 (64.5)	0.001
**Dialysis‐free survival**						
1‐year	0 (0)	6 (46.2)	33 (58.9)	18 (43.9)	155 (93.4)	0.001
5‐year	0 (0)	4 (30.8)	17 (30.4)	16 (39.0)	119 (78.3)	0.001
10‐year	0 (0)	3 (23.1)	14 (25.0)	14 (34.1)	79 (71.8)	0.004

*Note:* Results are presented with absolute numbers (*n*) and percentages in (). Percentages may not add up to 100% due to rounding.

**TABLE 5:** Multivariate Cox‐regression analysis regarding the risk of losing insulin‐independence post‐transplant for eras 3–5.

Variables		HR	95% CI	*p* value
Recipient age		1.03	1.00–1.07	0.43
Recipient gender	Male	Reference		
	Female	1.09	0.68–1.74	0.725
Recipient BMI		1.02	0.93–1.12	0.733
Donor age		1.01	0.99–1.02	0.464
Donor gender	Male	Reference		
	Female	1.26	0.81–1.97	0.312
Dialysis duration		1.00	1.00–1.00	0.385
Immunosuppression	Azathioprine	0.42	0.17–1.09	0.075
	Azathioprine + Ciclosporin	0.42	0.17–1.09	0.075
	Mycophenolate + Ciclosporin	0.29	0.02–3.86	0.349
	Mycophenolate + Tacrolimus	0.13	0.02–0.72	0.02
Induction therapy	Thymoglobuline	Reference		
	Basiliximab	0.91	0.31–2.65	0.862
Steroids < 5 days	>5 days	Reference		
	<5 days	0.51	0.16–1.58	0.241
Cold ischemic time		1.00	1.0–1.0	0.777
Era	3 (external drainage)	Reference		
	4 (bladder drainage)	0.55	0.30–1.02	0.058
	5 (enteric drainage)	0.72	0.23–2.28	0.575

*Note:* Events: 89; Global *p* value (Log‐Rank): 3.2865e‐07. AIC: 755.96; Concordance Index: 0.75. Results are presented as hazard ratios (HR).

Abbreviations: BMI, body mass index; CI, confidence interval; HR, hazard ratio.

## 4 Era 1: 1973–1976: First SPK—Era of Initial Experience

The first PT at the University of Zurich in 1973 was the first PT ever performed in Europe. Within this first era, four PTs were performed, all were SPK, with whole‐organ duodenum‐pancreas grafts. In the first three cases, surgical access was via bilateral lumbotomy incisions and retroperitoneal placement of pancreas grafts to the left, followed by kidney grafts to the right. In one patient median laparotomy was performed. Pancreas grafts were anastomosed via the donors` celiac trunk to the common iliac artery. Venous drainage was via grafts’ portal vein to the common iliac vein. Exocrine drainage was enteric, via Roux‐Y anastomosis. In the first three cases, a preceding surgery was performed some weeks before to prepare the Roux‐Y loop for later completion at the time of transplantation. The fourth PT was performed with a single surgery. Immunosuppression consisted of azathioprine and steroids. Median cold ischemia time (CIT) was 270 min, and Collins solution was used for cold flush and storage. All pancreases were from DCD donors, with a median donor age of 21 years. All recipients suffered from severe postoperative complications and died within a few weeks after transplantation, either from septic shock (*n* = 2) or myocardial infarction (*n* = 2). Longest patient survival was 111 days from transplantation. Pancreas graft function was limited, with only two patients achieving a short time of insulin freedom, one with a maximum length of 6 weeks. This patient lost both grafts for severe rejection, underwent multiple reoperations, and finally died from septic shock. Median follow‐up was correspondingly short, with a median of 1.5 months (IQR 0.7–3.6 months). This initial era was characterized by major innovations and anticipated some later developments, such as the use of the whole organ pancreas with enteric drainage. However, the overall poor results and high mortality rate led to a pause in the PT program until 1980.

## 5 Era 2: 1980–1983: Experimental era

With the restart of the PT program in 1980, only segmental pancreas grafts (Figure S1) were transplanted. A total of 13 PTs were performed within this era. All organs were from DBD donors, with a median donor age of 24 years. Arterial anastomosis was via the external or common iliac artery. The portal vein was drained systemically via the external iliac vein or vena cava. Pancreatic ducts were either left open or occluded. Duct occlusion was either by ligation or, in a few cases, additionally with Ethibloc (Ethicon, Norderstedt, Germany). In some cases, an omental patch was used for additional duct sealing. Immunosuppression was with azathioprine and long‐term steroids. Cyclosporin was first used in 1983, with the last segmental PT with duct ligation and block. Median CIT was 510 min, and Eurocollins was the preferred storage solution.

The overall complication rate was 91.7% until discharge and 100% at 90 days after transplantation. The main reasons for mortality were infections (38%) and myocardial infarction (31%). At 1, 5, and 10 years, overall patient survival and insulin‐free survival were 46.2%, 46.2%, and 46.2%, and 30.8%, 30.8%, and 23.1%, respectively. Dialysis‐free survival was 46.2% after 1 year, 30.8% after 5 years, and 23.1% after 10 years. Median follow‐up was 27.6 months (IQR 0.0–312.6 months).

## 6 Era 3: 1983–1992: Externalized Pancreatic Duct Drainage

Fifty‐six segmental pancreatic grafts with transcutaneous duct drainage were performed. Pancreatic ducts were extraperitonealized, using duct drainage and fixation of the pancreas graft to the abdominal wall (Figure S2). Venous drainage was systemic via the external iliac vein or vena cava, as previously performed. Patients received cyclosporine, azathioprine, and long‐term steroids for maintenance therapy. All organs were from DBD donors, with a median CIT of 360 min, and Eurocollins was mainly used as the storage solution. Major causes of death were cardiac events and infections (33% each). In selected cases (*n* = 23), an additional catheter was inserted via the hepatic or gastric artery stump of the splenic/celiac arterial graft and externalized percutaneously. This was used for continuous heparinization of the pancreas graft, with the aim of minimizing thrombotic complications. Of these, almost half of the patients suffered from severe bleeding complications (*n* = 11, 47.8%), requiring relaparotomy.

At 90 days, the overall complication rate was 100%, with a median CCI [25] of 58.4. At 1, 5, and 10 years, overall patient survival and insulin‐free survival were 73.5%, 51%, and 44.9%, and 34.5%, 25.5%, and 16.4%, respectively. Dialysis‐free survival was 58.9% after 1 year, 30.4% after 5 years, and 25% after 10 years. Median follow‐up was 69.5 months (IQR 0.5–475.7 months).

## 7 Era 4: 1992–1996: Bladder Drainage

Forty‐one PTs were performed with bladder drainage. All organs were transplanted as whole‐organ pancreas grafts, en bloc with adjacent duodenum, through a median laparotomy. Here, for the first time, a donor iliac Y‐graft was used, connecting the graft's superior mesenteric and splenic arteries. The Y‐graft was then anastomosed to the recipients’ common iliac artery. Venous drainage was systemic via the grafts` portal vein into the inferior vena cava. All organs were from DBD donors, with a median donor age of 35.5 years. Induction was by ATG, maintenance therapy with cyclosporine, azathioprine, and long‐term steroids.

Overall complication rate after 90 days was 97.5%, with a CCI of 53.1. The major cause of death was infection (22%). The main types of infection comprised urinary tract and wound infections. Fifteen (37%) pancreas grafts were later converted to enteric drainage, mainly for persisting urinary tract infections. At 1, 5, and 10 years, overall patient survival and insulin‐free survival were 69.7%, 60.6%, and 51.5%, and 41.5%, 34.1%, and 31.7%, respectively. Dialysis‐free survival was 43.9% after 1 year, 39% after 5 years, and 34.1% after 10 years. Median follow‐up was 192.9 months (IQR 0.8–376.5 months).

## 8 Era 5: 1996–2023: Enteric Drainage

A total of 166 enteric drained PT were performed in this last era, which brought substantial improvement in patient‐ and insulin‐free survival. The first enteric‐drained PTs were performed via Roux‐en‐Y reconstruction (*n* = 6) until 1997. From then on, duodeno‐jejunostomy became the standard anastomosis. Standard access was via median laparotomy, and pancreas grafts were placed “head‐up” to the right iliac axis. Arterial reconstruction was performed via iliac Y‐graft. This Y‐graft was later anastomosed to the right common iliac artery. Venous drainage was either systemic via the right common iliac vein or inferior vena cava, or in 49 cases, a portal drainage via the superior mesenteric vein was used. Kidney grafts were placed on the left iliac axis. Patients received ATG for induction. For maintenance therapy, Mycophenolate mofetil replaced azathioprine in 1997, and Cyclosporin was changed to Tacrolimus in 1998. In 2011, an early reduction of steroids (5‐day steroid tapering) was introduced. Since 2011, enteric‐coated mycophenolate (Myfortic) has replaced mycophenolate mofetil (CellCept) due to improved gastrointestinal tolerance.

At 90 days after PT, the overall complication rate was 84.9%. Median CCI at discharge and 90 days was 20.9 and 33.6, respectively. The main causes of death were infections and cerebrovascular events (20% each). Cardiac events were responsible for 5% of deaths. The era of enteric drained PT showed a significant decrease in overall complication rate after 90 days (*p* = 0.004), as well as CCI at discharge and 90 days (each *p* < 0.001), compared to the previous eras of PT. At 1, 5, and 10 years, overall patient survival and insulin‐free survival were 95.8%, 95.4%, and 82.1%, and 85.5%, 78%, and 64.5%, respectively. Dialysis‐free survival was 93.4% after 1 year, 78.3% after 5 years, and 71.8% after 10 years. Median follow‐up was 120.7 months (IQR 4.8–321.6 months).

We conducted a separate analysis for preemptive transplantations in era 5. We saw tendencies to reduced complications and better survival; however, we did not see significant differences. Additionally, we compared systemic and portal drainage within this era. Portal drainage showed longer operation time (360 min vs. 298 min; *p* < 0.001), but there were no differences in complications or survival, only a significantly better 5‐year dialysis‐free survival for the portal drainage group (91% vs. 72%; *p* = 0.008).

## 9 Multivariate Analysis

Insulin‐free survival was not influenced by donor and recipient characteristics, such as age, gender, and BMI. Similarly, the time of dialysis did not affect insulin‐free survival in these three eras. A tendency toward increased insulin‐free survival was observed following the introduction of MMF (HR 0.29 [95% CI: 0.022–3.86]; *p* = 0.349), and the combination of MMF and tacrolimus reached statistical significance (HR 0.13 [95% CI: 0.022–0.72]; *p* = 0.02). Early steroid withdrawal (<5 days) showed a tendency toward improved insulin‐free survival; however, this did not reach statistical significance (HR 0.51 [95% CI: 0.162–1.58]; *p* = 0.241). A separate multivariate analysis including only eras 4 and 5 showed a similar pattern, with the change to MMF and tacrolimus being the only significant factor. The analysis is provided in Table S1.

## 10 Discussion

This report accurately describes the historical developments and progress of PT at the Department of Surgery and Transplantation of the University Hospital Zurich over the last 50 years. A transition of complications from major to minor was observed over the course of five eras. The most remarkable advancements in this field occurred during the enteric drainage era. The reasons for the substantial improvements over time are multifaceted. The technical breakthrough that played a pivotal role in this transformation was the shift in pancreatic duct management to bladder‐ and enteric drainage. Subsequent innovations in immunosuppression and perioperative management further refined the field, culminating in the transformation of PT from an experimental procedure to a highly efficient, standardized, and well‐established transplantation. This analysis underlines the importance and value of PT. Although great advances in diabetes therapy have been achieved within the last decades, PT remains, next to islet transplantation, the only causal diabetes therapy to achieve complete insulin independence and improve patients` survival and quality of life. According to United Network of Organ Sharing (UNOS) data, patient survival at 1 and 5 years after deceased donor PT exceeded 95% and 83% [18]. Results of PT in Switzerland even surpassed these, with an overall patient survival of 100%, 97.8%, 92.4%, and insulin‐free survival rates of 89.5%, 81.5%, 78.3%, at 1, 5, and 10 years after primary SPK in the 21st century [19]. However, today's success and excellent outcome of PT were initially overshadowed by less successful early years, with high rates of complications and mortality. PT in the 1970s and early 1980s was dominated by technical difficulties, primarily originating from problems in the management of pancreatic duct drainage. The first 10 reported cases from 1966 to 1969 at the University of Minnesota showed a median survival of only 3.5 months, with ranges between 1 week and 11 months [6]. Although such results may sound devastating today, back then they were celebrated as great successes. In the early days of PT, recipients’ risk of mortality from T1D was estimated to equal the risk of the surgery. The early years of PT at the University of Zurich showed similarly poor results. The technique of SPK with whole organ pancreas‐duodenal grafts, systemic endocrine drainage, and enteric exocrine drainage was highly innovative in those days. Most likely, complications arose from severe rejection due to insufficient immunosuppression in the early days. The initial difficulties and the only short‐lasting success led to a break and interruption of the PT program for almost 4 years. The transplant program, however, continued with remarkable innovation. In 1978, Largiadér et al. performed the first simultaneous kidney‐ and intrasplenic islet allotransplantation worldwide [26]. The recipient had normal blood glucose levels without the need for exogenous insulin 1 year after surgery [26]. This success boosted the revival of PT. In 1980, the PT program was restarted with segmental PTs with different pancreatic duct managements, including duct ligation, duct occlusion, or percutaneous duct drainage (Figure S1, S2). However, success rates were again limited by severe complications, mainly caused by pancreatic fistulas, with a negative impact on patient‐ and graft survival. Nevertheless, most of the functional failures of transplants had been caused by postoperative vascular thrombosis or intestinal leaks, sometimes requiring multiple reoperations (Figure S3). Complications from the exocrine pancreas were reduced by postponement of percutaneous duct occlusion, using prolamine. The prevention of thrombosis via the described percutaneous continuous heparinization led to serious complications, especially severe hemorrhage, with the necessity of relaparotomy in every second PT. Under these circumstances in the late 1980s, PT was still considered experimental, and there was serious controversy and debate about whether the benefits outweighed the potential risks.

During this period, several other transplant centers published their first experiences with PT [7, 27]. Since then, novel surgical techniques, along with more effective immunosuppression and improved postoperative management, have brought notable progress, with decreasing complication rates and improved long‐term outcomes [10, 11, 12]. The introduction of cyclosporine led to a reduction of rejection rates [28]. However, long‐term steroid therapy remained one of the backbones of our immunosuppressive protocol, due to the fear of kidney rejection in SPK. Steroids remained as long‐term therapy until 2011, when the protocol was changed to early steroid withdrawal 5 days after transplantation. Between the late 1980s and early 1990s, PT shed its experimental status and established itself slowly as a more routine and standardized treatment.

The first major technical improvement was made from 1992 on, with the change to bladder drainage [29]. Draining the exocrine pancreas into the bladder allowed for monitoring the amylasuria and facilitated access for graft biopsies [30]. A decline in amylasuria activity represented a practical tool for the timely diagnosis of graft rejection. This led to a significant decrease in immunological graft loss [31]. Since this era, pancreas grafts have again been used as whole organs. Later, the use of the duodenal segment bladder technique even resulted in another drop in early complication rates. However, urinary tract infections [32] and metabolic acidosis from excessive bicarbonate loss complicated this technique [33]. This led to the implementation of enteric drainage techniques and later resulted in high conversion rates from the bladder to enteric drainage [34]. The conversion rate from the bladder to enteric drainage was 37% in our cohort, which corresponds to the literature. It is reported that up to 41% of patients with bladder drainage needed conversion to enteric drainage within 10 years, when precious grafts could be lost due to conversion [12, 35].

One major upgrade was the introduction of enteric‐drained whole‐organ pancreaticoduodenal transplants by Starzl et al. in 1984 [36]. This was similar to the originally described technique by Lillehei [37], and brought a major shift from bladder to enteric drainage in the late 1990s. However, initially, enteric drainage resulted in a high rate of duodenal leaks and worse results than bladder drainage [38]. Importantly, this was frequently associated with histologic evidence of acute rejection. This problem could be overcome from the mid‐1990s with the introduction of Tacrolimus and Mycophenolate mofetil [34, 39], not only reducing rejection‐mediated graft loss [40] but also leading to a severe decline in graft thrombosis, intra‐abdominal infections, and the rate of relaparotomy [41]. Our department changed to enteric drainage in 1996. This was, in principle, a return to the previously used enteric drainage technique used at the very beginning of PT by Largiadér et al. A simplified technique of exocrine drainage via side‐to‐side anastomosis of the graft duodenum and the proximal jejunum, without the need for Roux‐en‐Y loop, or pancreatic duct catheter, was introduced by the Stockholm group (Groth and Tyden) and boosted the success of intestinal drainage [42]. Between 1994 and 1997, 1‐year patient‐ and graft survival rates for SPK exceeded 90% and 80%, respectively [12, 18].

The overall rate of complications is still noteworthy but was significantly decreasing over the different eras and was in line with the literature [43, 44, 45, 46, 47]. This is all the more remarkable as donors and recipients become increasingly older and more comorbid [48]. T1D is a leading risk factor for cardiovascular disease, and high rates of asymptomatic coronary artery stenosis have been reported in diabetic patients with ESKD [49]. This is why recipients of PT are at high risk for cardiac events [50], and up to now, cardiovascular disease remains the most common cause of death after SPK [51]. This is in line with our analysis, in which cardiac events were the main cause of early postoperative mortality, followed by infectious complications. Overall cardiac mortality could be reduced from initially 50% to 5% in the enteric‐drained era. The most important measure to improve patient survival after PT was the implementation of a structured preoperative cardiac workup. Nowadays, elective coronary angiography is performed in all patients on dialysis before waitlisting. In patients with residual kidney function, a cardiac positron emission tomography scan is performed before waitlisting.

When the overall number of 280 PTs at the University Hospital of Zurich is considered, it appears to be relatively low in comparison to high‐volume centers. However, our transplant center has extensive experience and expertise and is the largest transplant center in Switzerland, performing all types of transplants. Notably, we have a high‐volume kidney transplant program, performing around 120 transplants annually, and patients after SPK are cared for by the same team. Furthermore, Switzerland's demographics can well explain the limited numbers. In a small country like Switzerland, which had only about 6.5 million people in 1973 and 8.6 million in 2020, only 0.6% of the population suffers from type 1 diabetes mellitus. With an extremely dense healthcare system and ever‐improving diabetes therapies and patient management worldwide, the number of people who actually need a pancreas transplant is limited. Notably, we are proud that our center has consistently performed a stable number of PTs and is still active, despite the global decrease in PTs, and while many other centers have completely stopped their programs in the last years and many more are expected to follow.

This study has some limitations. First, many patients from the early years of PT were lost to follow‐up, or the data were incomplete. Second, we could not fully account for potentially significant changes in post‐PT treatments over the different eras, including changes in preoperative assessment and treatments, possibly affecting donor and recipient selection. Third, the number of PTs within the early eras was too low to draw significant conclusions.

## 11 Conclusion

The history of PT at the University Hospital of Zurich spans over 50 years and is a story of continuous improvement, innovation, and progress. Major technical developments, paired with novelties in immunosuppression, led to the distinction of different eras of PT, characterized by significantly reduced complications and improved patient and graft survival over time. Over the decades, PT has evolved to a highly efficient treatment and remains the only causal diabetes therapy to reliably achieve total insulin independence.

## Author Contributions

Concept/design: Michael C. Frey, José Oberholzer, and Fabian Rössler. Data collection: Michael C. Frey and Sandro Hügli. Statistics: Sandro Hügli. Data analysis/interpretation: Fabian Rössler, Michael C. Frey, and Sandro Hügli. Drafting article: Michael C. Frey, José Oberholzer, and Fabian Rössler. Critical revision of article: Michael C. Frey, Sandro Hügli, Olivier de Rougemont, Kerstin Hübel, Thomas Schachtner, Elena Rho, Lukas Weidmann, Roger Lehmann, Jakob Nilsson, Lukas Frischknecht, José Oberholzer, and Fabian Rössler.

## Conflicts of Interest

The authors of this manuscript have no conflicts of interest to disclose.

## Supporting information




**Supporting File 1**: ctr70368‐sup‐0001‐SuppMat.docx

## Data Availability

Data is available upon reasonable request to the corresponding author.
