# Supplementary material for: Five Decades of Pancreas Transplantation at the University Hospital of Zurich—A Story of Continuous Improvement and Success
Source: Clin Transplant. 2025 Nov 5;39(11):e70368. doi: 10.1111/ctr.70368 (PMC12588550; doi:10.1111/ctr.70368)
Supplement: Supplementary file 1 — Supporting File 1: ctr70368‐sup‐0001‐SuppMat.docx [file CTR-39-e70368-s001.docx]

**Supplementary Material:**

**Supplementary image 1:** Simultaneous segmental pancreas- and kidney transplantation, with arterial and venous anastomosis to the common iliac vessels (original drawing by the transplant surgeon, Zurich, 4.7.1980).


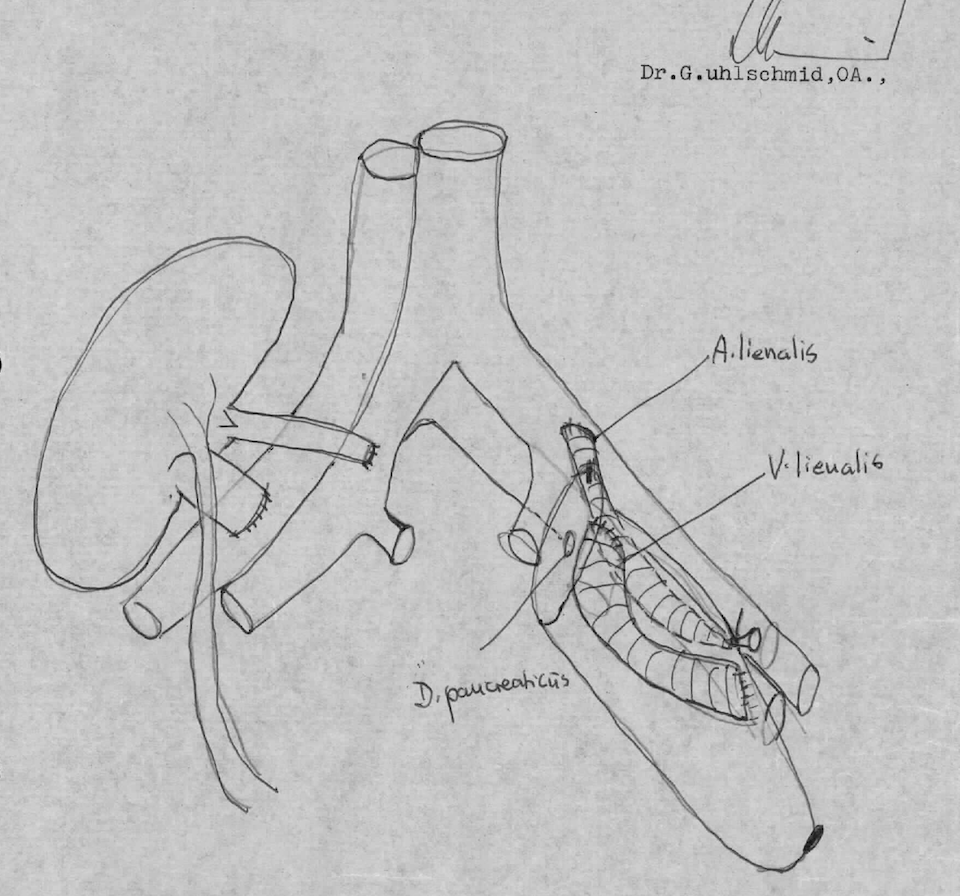


**Supplementary image 2:** Simultaneous segmental pancreas- and kidney transplantation, with externalized pancreatic duct drainage. Additionally, a catheter has been inserted via the hepatic or gastric stump of the grafts` arterial supply via the celiac trunk and splenic artery for continuous postoperative heparin infusion (original drawing by the transplant surgeon, Zurich, 13.09.1988).


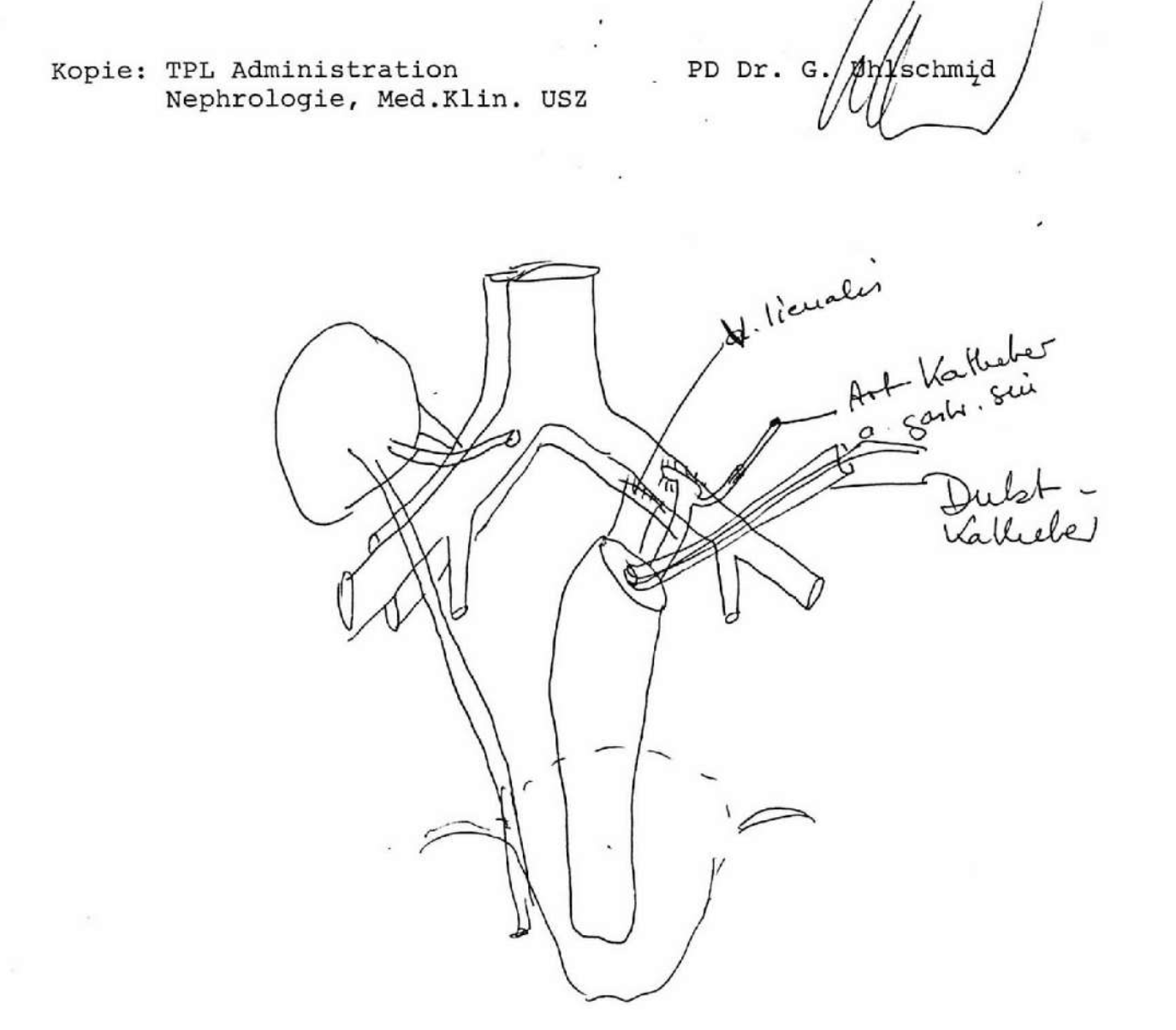


**Supplementary image 3:** Illustration of the abdominal drainage position, after relaparotomy for intraabdominal abscess formation following a segmental pancreas- and kidney transplantation with a complicated course (original drawing by the transplant surgeon, Zurich, 23.06.1986).

**
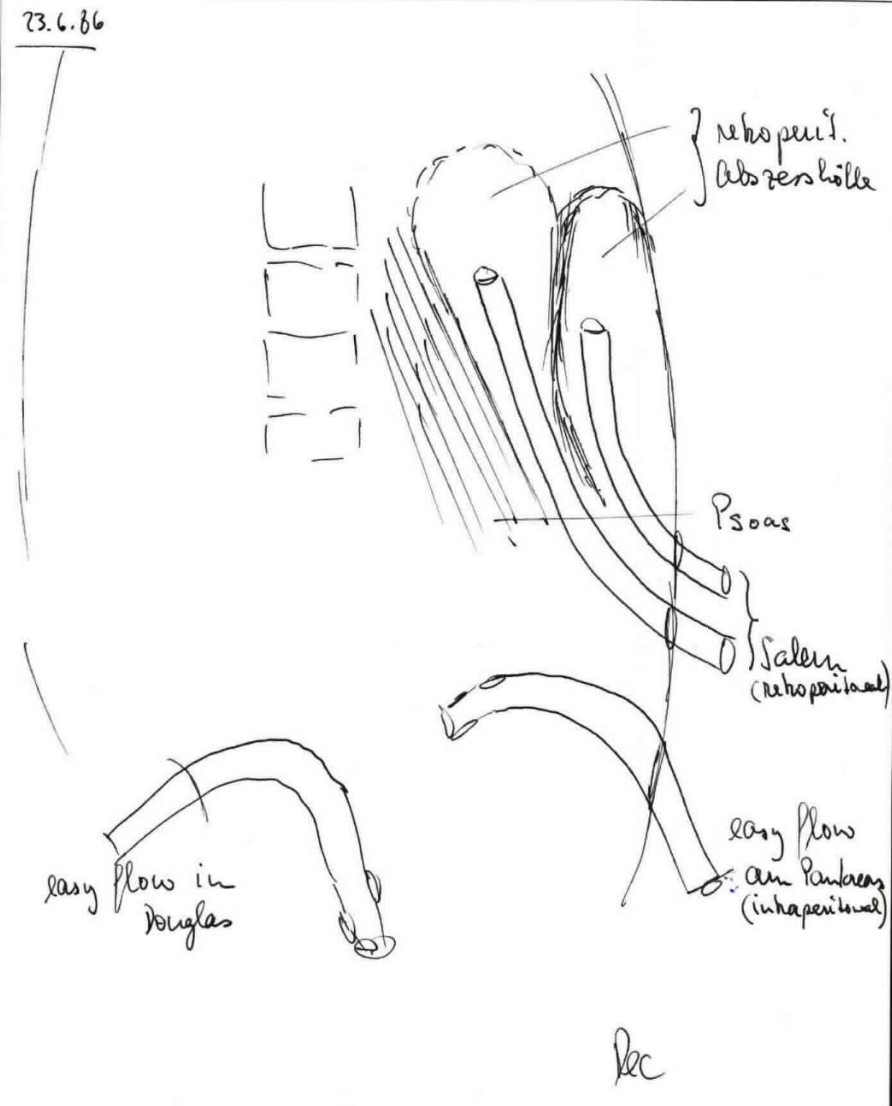
**

**Supplementary figure 1:** Number of pancreas transplantations per year****

**Supplementary table 1:** Multivariate cox-regression analysis regarding the risk of losing insulin-independence post-transplant for eras 4 and 5. Results are presented as Hazard Ratios (HR).


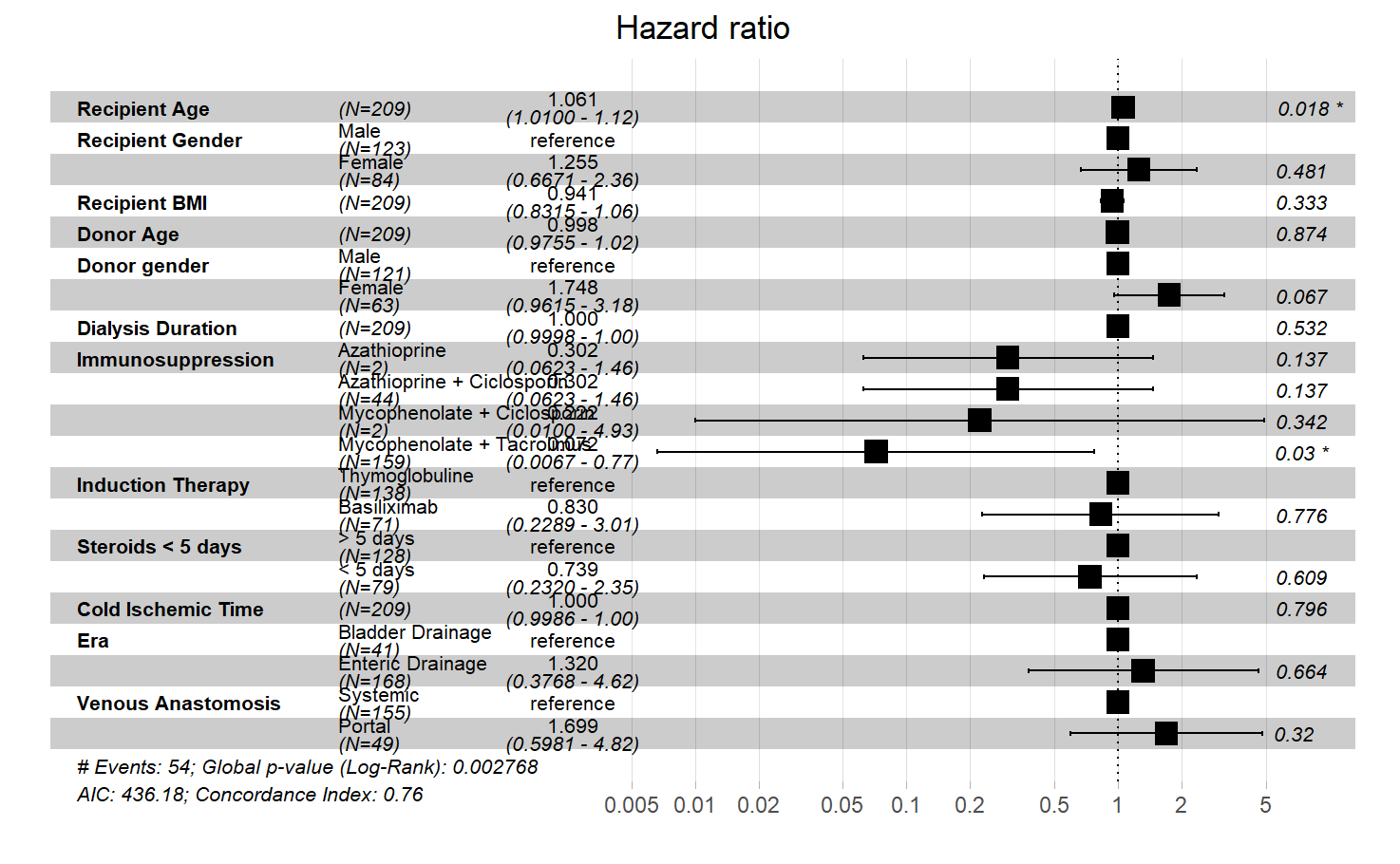


**Supplementary table 2:** Separate analysis of preemptive transplantations regarding patient and graft survival.

|  | **Overall**  **(n = 160)** | **Preemptive**  **(n = 47)** | **Dialysis**  **(n = 113)** | **p-value** |
| --- | --- | --- | --- | --- |
| **Overall survival** | | | | |
| 1-year | 153 (96) | 45 (96) | 108 (96) | 0.9 |
| 5-year | 119 (95) | 35 (95) | 84 (95) | 0.9 |
| 10-year | 83 (81) | 20 (77) | 63 (83) | 0.6 |
| **Insulin-free survival** | | | | |
| 1-year | 138 (86) | 41 (87) | 97 (86) | 0.8 |
| 5-year | 99 (79) | 31 (84) | 68 (76) | 0.4 |
| 10-year | 68 (65) | 18 (69)) | 50 (63) | 0.8 |
| **Dialysis-free survival** | | | | |
| 1-year | 150 (94) | 43 (91) | 107 (95) | 0.5 |
| 5-year | 114 (78) | 34 (79) | 80 (78) | 0.9 |
| 10-year | 75 (71) | 19 (73) | 56 (71) | 0.8 |

Categorical or ordinal scaled variables are presented with absolute numbers (n) and percentages in (). Percentages may not add up to 100% due to rounding.

**Supplementary table 3:** Pancreas – specific complications until discharge.

| **Variables** | **Era 1**  **(n = 4)** | **Era 2**  **(n = 13)** | **Era 3**  **(n = 56)** | **Era 4**  **(n = 41)** | **Era 5**  **(n = 166)** |
| --- | --- | --- | --- | --- | --- |
| **Complications** | | | | | |
| Graft pancreatectomy for venous thrombosis |  | 1 | 5 | 5 | 11 |
| Graft pancreatectomy for arterial thrombosis |  |  | 4 | 1 |  |
| Graft pancreatectomy for arterial and venous thrombosis |  |  | 4 |  |  |
| Relaparotomy with open thrombectomy pancreas |  |  |  |  | 2 |
| Graft pancreatectomy for bleeding |  |  |  |  | 3 |
| Relaparotomy for peripancreatic abscess / fluid collection |  | 2 | 17 | 2 | 11 |
| Relaparotomy for bleeding |  |  | 14 | 8 | 17 |
| Relaparotomy for graft duodenal leakage | 1 |  |  | 4 | 1 |
| Reoperation for wound complications | 2 |  | 2 | 3 | 6 |
| Relaparotomy without clear finding |  | 1 |  |  | 4 |
| Angiography and coiling of pancreatic aneurysm |  |  |  |  | 1 |
| Percutaneous drainage of peripancreatic abscess / fluid collection |  | 4 | 8 | 4 | 14 |
| Postoperative Ileus / Gastroparesis | 2 |  | 1 | 2 | 16 |
| Treatment for pancreas rejection | 2 | 5 | 23 | 23 | 13 |

Some patients developed more than one complication and multiple complications in the same patient were all counted separately.

**Supplementary table 4:** Separate analysis of patients and graft survival for systemic and portal drainage in Era 5.

|  | **Overall**  **(n = 166)** | **Systemic**  **(n = 119)** | **Portal**  **(n = 47)** | **p-value** |
| --- | --- | --- | --- | --- |
| **Overall survival** | | | | |
| 1-year | 158 (96) | 113 (96) | 45 (96) | 0.9 |
| 5-year | 124 (95) | 81 (96) | 43 (93) | 0.7 |
| 10-year | 87 (82) | 47 (78) | 40 (87) | 0.3 |
| **Insulin-free survival** | | | | |
| 1-year | 142 (86) | 103 (87) | 39 (83) | 0.6 |
| 5-year | 103 (78) | 68 (80) | 35 (74) | 0.5 |
| 10-year | 71 (65) | 40 (63) | 31 (66) | 0.8 |
| **Dialysis-free survival** | | | | |
| 1-year | 155 (93) | 111 (93) | 44 (94) | 0.9 |
| 5-year | 119 (78) | 76 (72) | 43 (91) | 0.008 |
| 10-year | 79 (72) | 41 (65) | 38 (81) | 0.069 |

Categorical or ordinal scaled variables are presented with absolute numbers (n) and percentages in (). Percentages may not add up to 100% due to rounding.
